# The Study of Zeolitic Imidazolate Framework (ZIF-8) Doped Polyvinyl Alcohol/Starch/Methyl Cellulose Blend Film

**DOI:** 10.3390/polym11121986

**Published:** 2019-12-02

**Authors:** Shaoxiang Lee, Yunna Lei, Dong Wang, Chunxu Li, Jiaji Cheng, Jiaping Wang, Wenqiao Meng, Meng Liu

**Affiliations:** 1 College of Environment and Safety Engineering, Qingdao University of Science and Technology, Qingdao 266042, China; 2 Shandong Engineering Research Center for Marine Environment Corrosion and Safety Protection, Qingdao University of Science and Technology, Qingdao 266042, China; 3 Shandong Engineering Technology Research Center for Advanced Coating, Qingdao University of Science and Technology, Qingdao 266042, China; 4 ASTUTE 2020 in Future Manufacturing Research Institute, College of Engineering, Swansea University, Swansea SA1 8EN, UK

**Keywords:** PVA/starch, composite membrane, metal-organic frameworks

## Abstract

ZIF-8 nanoparticle-doped polyvinyl alcohol (PVA)-S-MC films were prepared via casting method. The effect of different concentrations of ZIF-8 on the physical properties and structural characterization of the films were investigated. The results indicated that ZIF-8 could increase the water resistance and mechanical property of the membrane. Through FTIR, scanning electron microscope (SEM), atomic force microscope (AFM), and TGA analysis, it was found that ZIF-8 changed the phenomenon of macromolecule agglomeration and improved the thermal stability of the membrane. The breathable behavior of the film was also studied through oxygen permeability and water vapor permeability analysis. The result illustrated that the breathability of the film improved significantly by adding ZIF-8. The maximum reached when the weight ratio of ZIF-8 was 0.01 wt %. The property expands the application of PVA/starch blend film in the postharvest technology of fruits and vegetables.

## 1. Introduction

In the past decades, the excessive use of petroleum based plastics had generated amounts of non-degradable waste, which could cause severe impact on both marine and continental ecosystem, as well as the health of human beings and animals [1]. A sustainable, degradable, and economically viable material is hereby needed. Starch is one of the most promising materials for biodegradable polymers because of its low cost, abundance in nature, and availability. Some of the disadvantages of starch polymer, such as poor mechanical properties and water barrier, seriously hinder its applications. However, the shortcomings of starch films could be improved by blending other synthetic or composite materials. Among the great diversity of biodegradable materials, polyvinyl alcohol (PVA) is a potential material which possesses favorable mechanical property, excellent transparency, and thermal stability, as well as good gas barrier and water vapor permeability. PVA and starch have favorable compatibility because of the existence of hydroxyl in the molecules. Thus, PVA/starch blends have drawn widespread interest of researchers because of its nontoxic, membrane-forming, degradable and favorable mechanical property in recent years. Kanda prepared cassava starch/PVA membrane with high crystallinity and hydrophobicity. Luo found the *T*_m_ of corn starch/PVA membrane could be improved by gelatinization of starch [2]. The moisture sensitivity of starch/PVA membrane could be reduced by crosslink with sodium hexametaphosphate [3]. The PVA/starch membrane also has excellent physical properties because of the ageing of starch was inhibited [4].

To further improve the functionality of PVA/starch membrane, many modification strategies have been developed. The nano-materials were reported that could effectively improve the strength, thermal stability, hydrophilicity, and antibacterial properties of the PVA/starch membranes. Nooshin and some researchers claimed that the mechanical properties of PVA/starch membrane were enhanced with the addition of cellulose [5,6,7,8]. Hatami improved thermal stability of the PVA membrane by incorporating nanosilica while Wu introducing graphene oxide [9,10]. Jahan and Wang revealed the moisture uptake decreased by adding cellulose nanocrystal and clay, respectively [11,12]. Some researchers [10,13,14] introduced nanotitania and silver nanoparticles into PVA/starch membrane, the results demonstrated those nanomaterials have positive effects on antibacterial properties.

Mental-organic frameworks (MOFs) possess many preeminent properties such as high specific surface area, high porosity, antibacterial properties, adjustable pore size, and selective permeability for gases, as well as outstanding thermal and chemical stability [15,16,17,18,19,20]. Frederik employed trifluoroacetic acid and HCl during the synthesis of UIO-66, the crystallinity and pore size were raised with partial substitution of terephthalates by trifluoroacetate [21]. Jianwei et al. found the particle size increased with the addition of formic acid while agglomeration reduced [22]. Bingchen achieved their best CO_2_ separation ability through controlling the size and morphology of ZIF-8 by adjusting the reaction time [23]. Mohd enhanced both CO_2_ permeability and CO_2_/CH_4_ selectivity of 6FDA-DAM membrane by introducing Zr-MOF [24]. Hulya reported that doped different metal ions such as Cu, Co in MOF could improve gas permeability in different content [25]. However, there were rarely related reports concern about MOFs PVA/starch film.

Zeolitic imidazolate frameworks (ZIF-8), are subspecies of metal-organic frameworks with zeolites isomorphs which are widely used in the gas separation. Therefore, the aim of the present study was to develop a water-soluble and degradable PVA/starch/cellulose blend film doped by ZIF-8 via solution casting method. PVA and starch were employed as mainly membrane-forming materials, and methyl cellulose was introduced to reinforce and mitigate the poor mechanical properties. 1,4-butanediol was used as plasticizer to subvert the structure of high molecules and weaken the mutual interaction between the raw materials. A certain amount of ZIF-8 nanoparticles were added to improve the breathability and physical properties of the composite membrane. Besides, microstructure, oxygen permeability, hydrophilic, mechanical property as well as thermal analyses of the modified membrane were evaluated.

## 2. Materials and Methods

### 2.1. Materials

Polyvinyl alcohol (PVA) (degree of polymerization: 1700, degree of hydrolysis: 87%–89%), were purchased from Aladdin reagent Shanghai Co., Ltd. (Shanghai, China); corn starch (Sinopharm Chemical Reagent Co., Ltd. Beijing, China); methyl cellulose-M450 (Tianjin Damao Chemical Reagent Factory, Tianjin, China); 1,4-butanediol (Tianjin Beilian Fine Chemicals Development Co., Ltd. Tianjin, China); methanol (Sinopharm Chemical Reagent Co., Ltd. Beijing, China); zinc nitrate hexahydrate (Tianjin Damao Chemical Reagent Factory, Tianjin, China); 2-methylimidazole (Aladdin reagent Shanghai Co., Ltd. Shanghai, China). All the chemicals were analytically pure. The deionized water (Millipore Milli-Q) was used throughout.

### 2.2. Preparation of ZIF-8 Nanoparticle and PVA/Starch Blend Film

ZIF-8 nanoparticles were synthesized using the solvothermal method. Total of 0.297 g zinc nitrate hexahydrate and 0.66 g 2-methylimidazole were each dissolved in 11.3 g methanol solution. The solutions were sonicated at room temperature until zinc nitrate hexahydrate and 2-methylimidazole were sufficiently dissolved in methanol before they were mixed up. The ZIF-8 growth was conducted at 40 °C with stirring for 2 h. The ZIF-8 nanoparticles were dried at 120 °C after triple cleaning and centrifuged with methanol.

The PVA/starch/methyl cellulose blend films were prepared by the solution casting method. The constant weight ratio of PVA and starch (7:3) were used throughout the whole experiment. A total of 7 g PVA and 3 g starch were dissolved in 70 mL 90 °C deionized water with constant stirring for 1 h. 10 g 1, 4-butanediol was utilized as the plasticizer. Different weight ratios of cellulose (1, 3, 5, 7 wt. %, *w*/*w* of PVA/starch blend) were added into the homogeneous mixture after it cools down to 70 °C in order to increase the solubility of starch and cellulose in water. For the preparation of PVA/starch/MC/ZIF-8 films, the ZIF-8 particles (0.01, 0.05, 0.09, *w*/*w* of PVA/starch blend) were added into the deionized water for 60 min ultrasonic dispersion. The ZIF-8 solution was then mixed with the blend at 90 °C and stirred vigorously for 12 h until the formation of a homogeneous solution. Finally, the nanomaterial composite solution was cast onto the PTFE plate. The thickness of the polymer film was maintained by a micrometer adjustable film applicator (BGD 209/2, Guangzhou Standard Geda Laboratory Instrument Co., Ltd. Guangzhou, China); the average thickness of each film is listed in Table 1. The films were preserved in a desiccator with constant temperature and humidity after dried in the oven at 80 °C for 20 min and 110 °C for 10 min.

### 2.3. Nanostructure Characterization

FT-IR spectra of the ZIF-8 nanoparticles and blend films were recorded at room temperature using IRAffinity-1 spectrometer (Bruker Corporation Blairica, Massachusetts, USA) attached to the universal ATR accessory over the wavenumber range from 4000 to 400 cm^−1^ and 2 cm^−1^ resolution. The films were mounted directly in the sample holder while the ZIF-8 nanoparticles were mixed with KBr powder where the pure KBr baseline was subtracted from the spectra. The XRD pattern of ZIF-8 nanoparticles were tested by X-ray diffractometer (Shimadzu Corporation, Tokyo, Japan) with a nickel-filtered Cu Kα radiation beam (40 mA and 40 kV). The analysis was scanned from 2θ = 3° to 50° with 1°/min scanning speed and 0.05° scan amplitude at ambient temperature. The Jade 6.0 XRD pattern processing software was utilized for statistical analysis.

### 2.4. Morphology Analysis

The morphology of ZIF-8 nanoparticles, surface and cross-section of the film samples were observed with a JSM-6700F scanning electron microscope (SEM) (JEOL, Japan) operated at a voltage of 5.0 or 10.0 kV. The AFM measurements were examined by MULTIMODE8 (Brook Technology Co., Ltd. NASDAQ, USA). The membrane samples were placed on optical glass at ambient temperature with a silicon probe with a nominal tip radius of 2 nm and a resonant frequency of 70 kHz. The scanning rate and area were 0.9 Hz and 20 µm × 20 µm respectively.

### 2.5. Mechanical Properties

The tensile strength (TS) and elongation at break (EB) of the films were measured with A1-7000M tensile tester (High Speed Rail Technology Co., Ltd. Taiwan, China) according to the ASTM standard method D882-12. The 4 mm × 75 mm dumbbell-shaped samples were cut from each prepared film and mounted between the grips of the machine. The initial grip separation was set to 50 mm/min.

### 2.6. Thermo-Gravimetric Analysis

The thermal properties of the PVA/starch/MC/ZIF-8 blend films were measured using a SDT-Q600 thermal analyzer (TA Instruments, Newcastle, Delaware, USA). The samples of about 10 mg were conditioned in an alumina crucible and heated from 25 to 800 °C at the rate of 10 °C/min with 50 mL/min nitrogen flow.

### 2.7. Contact Angle

The hydrophilic of the film was estimated by optical contact angle method using a Digidrop DX (GBX, Stuttgart, Germany). About 2 μL Millipore water droplets were put on the film surface with a Micro syringe. The contact angle between the baseline of the drop and the tangent at the drop bound-aryvalue were measured by a camera MV-50. At least three measurements were made for each film sample and the average was calculated.

### 2.8. Water Vapour Permeability (WVP)

The method carried out by Muhammad Salman Sarwar [14] was simulated to examine the WVP of the film samples. Total of 10 mL of deionized water was poured into a beaker with a diameter of 29.5 mm which was covered by film samples and tightened with Teflon tape. The weight of the beakers was measured and then placed in an oven for 24 h at 40 °C. After 24 h, the beakers were withdrawn from the oven and weighed again. Changes in the weight of the beaker were recorded as a function of time. Water vapor permeability was calculated by Equation (1)

(1) $$\mathrm{WVP}\;=\;\frac{Wi-We}{A*T}(g/m^{2}\;h)$$

where *W_i_* is the initial weight of beaker; *W_e_* is the weight of beakers at time *T*; *A* is the transmission area of membrane; *T* is 24 h.

### 2.9. Water Solubility (WS)

The water solubility was characterized by recording the dissolve time of the film samples in the deionized water. The film samples were cut into 15 mm × 15 mm and dried in the desiccator containing calcium chloride until the constant weight reached. A 5-mm cross was marked on the center of each film sample. The time which the cross disappear as the film sample immersed in deionized water was defined as water solubility time of the film.

### 2.10. Oxygen Permeability (OP)

Oxygen permeability was measured by using the constant volume-variable pressure method with Y310 membrane permeability testing machine (Guangdong Standard Packaging Equipment Co., Ltd., Guangzhou, China) at 23 °C and 53% RH [12]. The effective contact area during the measurements was 50.25 cm^2^.

### 2.11. UV-Visible Spectroscopy

The optical clarity of the PVA-S-MC film and PVA-S-MC-ZIF-8 nanocomposite films was measured by the Perkin-Elmer Lambda25 UV/Vis spectrophotometer (PerkinElmer, New castle wilmilton, Delaware, USA). The samples were cut in rectangular shapes. The scan range was 200–800 nm and step size was 2 nm.

### 2.12. Statistical Analysis

Statistical difference in the properties of different samples were analyzed with ANOVA via SPSS (version 22.0, SPSS Inc., Chicago, IL, USA). 

## 3. Results and Discussion

### 3.1. Characterization of ZIF-8 Nanoparticles and PVA/Starch/MC/ZIF-8 Blend Films

**Figure 1:**
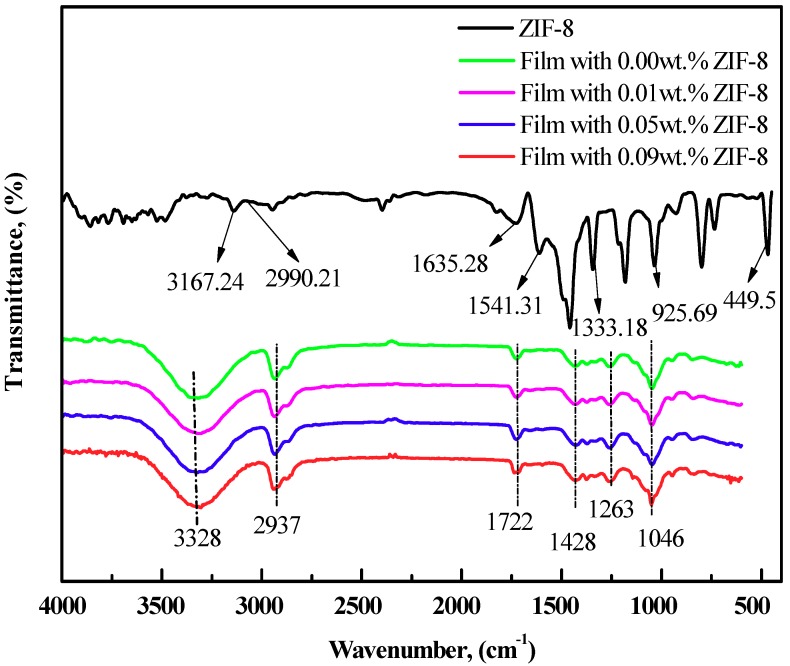
FTIR spectrum of ZIF-8 nanoparticles and polyvinyl alcohol (PVA)/starch/MC/ZIF-8 blend films.

The FTIR spectrum of the synthesized ZIF-8 nanoparticles and PVA/starch/MC/ZIF-8 blend films are depicted in Figure 1. In terms of the ZIF-8 nanoparticles, the absorption bands at 3167.24 and 2990.21 cm^−1^ corresponded to C–H stretching of C=C and –CH_3_, respectively. The bands at 1541.31 and 1635.28 cm^−1^ were attributed to C=N and C=C stretching of imidazole ring while the sharp band at 449.50 cm^−1^ was assigned to N–Zn stretching. The band at 1333.18 cm^−1^ was related to the C–H bending vibration of –CH_3_ and the band at 925.69 cm^−1^ was associated to the N–H swing. The peak position and intensity are well agreed with the previous reports [18,26,27,28,29]. Then different concentrations of ZIF-8 nanoparticles were added into the PVA/starch/MC blend. ZIF-8 nanoparticles have good affinity and compatibility with the organic matrix because of the presence of the organic imidazole ring in the molecule. Besides, the existence of nitrogen in ZIF-8 molecule could form a hydrogen bond with hydrogen in the polymer matrix, which accelerates the film formation. It can be seen that the infrared absorption peak at the wavelength from 3500 to 3000 cm^−1^ was the characteristic absorption peak of hydroxyl stretching vibration caused by the stretching frequency of PVA and water. With the increase of the ZIF-8 concentration, the O–H stretching vibration band shifted to the high-frequency region and corresponding bands of the membrane became narrower and stronger. The peaks near 2937 and 2935 cm^−1^ were related to the C–H stretching of C=C, –CH_3_, and –CH_2_. The infrared absorption peak near the wavenumber 1722, 1428, 1264 cm^−1^ were contributed by the C=O stretching, C–H bending, and C–C stretching respectively. The peak near the wavenumber 1046 cm^−1^ attributed to C–O stretching of PVA/starch/MC5% and C–N stretching of ZIF-8 [10,30,31]. As the concentration of ZIF-8 increased, the hydrogen bond formed by the reaction between ZIF-8 and films enhanced the degree of polarization of the chemical bond, making the absorption peak stronger.

**Figure 2:**
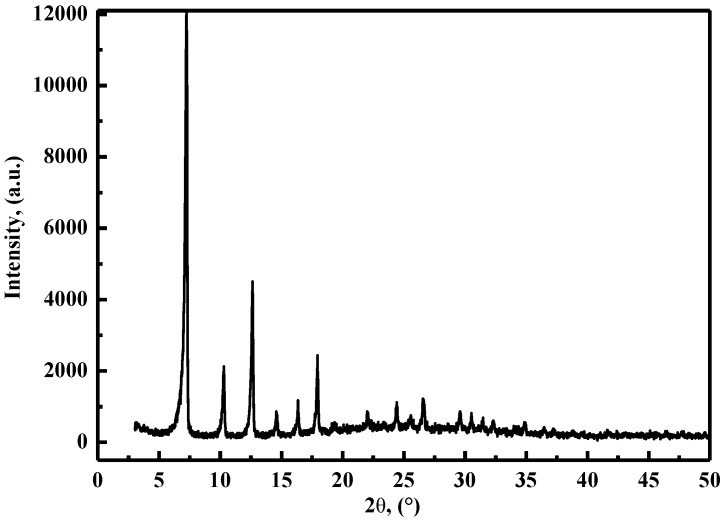
XRD pattern of ZIF-8 nanoparticles.

**Figure 3:**
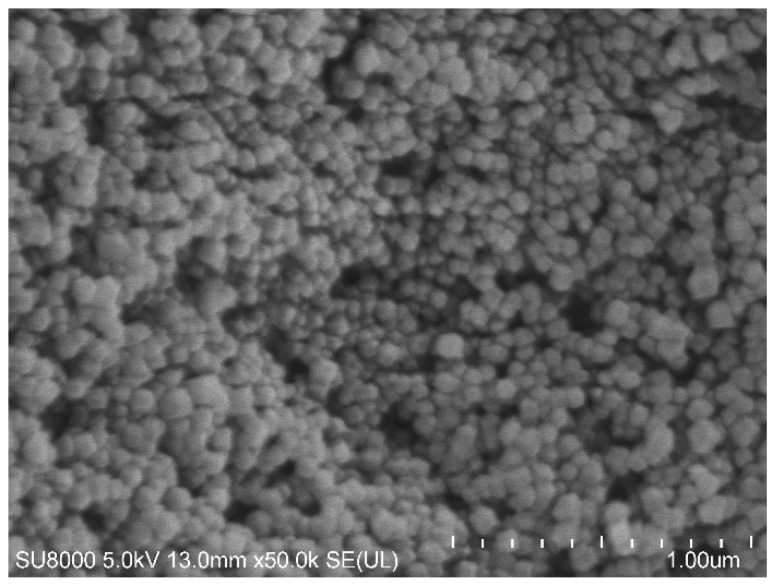
Scanning electron microscopy (SEM) image of ZIF-8 nanoparticles.3.2 Film morphology analysis.

The crystalline structure of the synthesized ZIF-8 nanoparticles was investigated by XRD analysis as demonstrated in Figure 2. It can be found that the characteristic diffraction peaks of ZIF-8 at 2θ = 7.24°,10.30°,12.64°,14.59°,16.36°,17.94°, 24.42°, and 29.58° [18,26,28,29,32,33,34]. The result indicated that the ZIF-8 nanoparticle has formed a favorable crystal structure which has great potential to improve the mechanical support and structural stability of the PVA/starch blend films. The morphology of ZIF-8 nanoparticles is shown in Figure 3. It can be seen from the SEM pictures that the nanoparticles are hexagonal in shape and the average diameter is around 100 nm, which is similar to the literature dates reported by [18,29,32,35].

### 3.2. Morphology Analysis of Films

**Figure 4:**
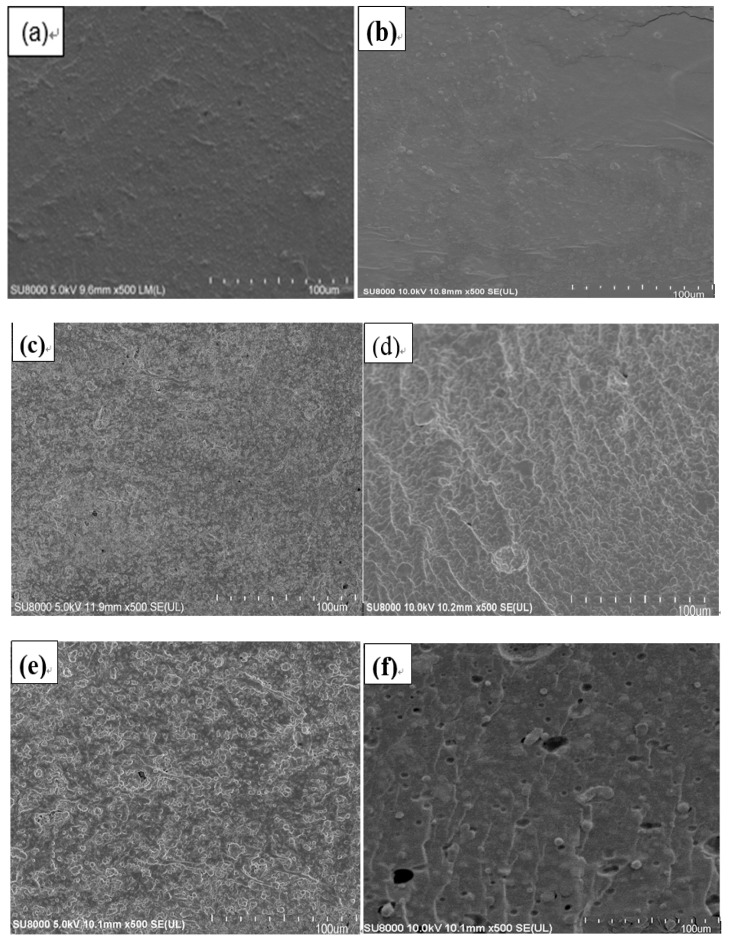
SEM images of PVA/starch/MC blend films with different concentrations of ZIF-8 nanoparticle: (**a**,**b**) surface and cross-section of film with 0.00 wt. % ZIF-8; (**c**,**d**) surface and cross-section of film with 0.01 wt. % ZIF-8; (**e**,**f**) surface and cross-section of film with 0.09 wt. % ZIF-8.

The microstructure, spatial distribution, and dispersion of ZIF-8 in the PVA/starch/MC5% were observed through a scanning electron microscope (SEM) and an atomic force microscope (AFM). As shown in Figure 4, the blend film without ZIF-8 has smooth surface (a) and cross-section (b), which reflected the satisfactory dispersion and compatibility of the high polymer materials. Both of the surface (c) and cross-section (d) morphologies of films became rougher with the addition of ZIF-8 nanoparticles. The results agreed with the previous reports [18,26,28,36]. Although the roughness of the films increased with concentration of ZIF-8, the distribution of the nanoparticles in the composite became better simultaneously. The uniform dispersion of ZIF-8 could be attributed to the intrinsic positive charge of 2-methylimidazole after protonation. So that the ZIF-8 nanoparticles repelled each other by strong electrostatic interaction than aggregated [33]. Besides, the PVA/starch/MC and ZIF-8 nanoparticles could disperse in the same solvent, which is beneficial for the evenly mixing of ZIF-8 into the blend solution [27].

**Figure 5:**
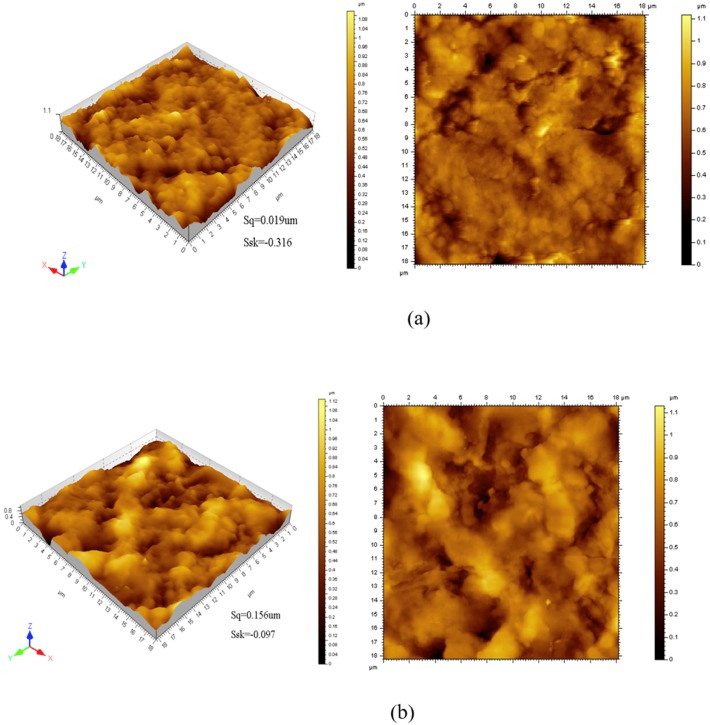

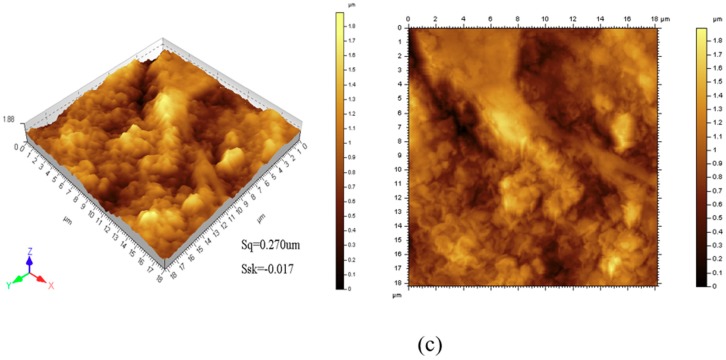
AFM images of PVA/starch/MC blend films with different concentrations of ZIF-8 nanoparticles: (**a**) the film with 0.00 wt. % ZIF-8; (**b**) the film with 0.01 wt. % ZIF-8; (**c**) the film with 0.09 wt. % ZIF-8.

The atomic force microscopy images are exhibited in Figure 5. The 3D images of the films show a rough structure with valleys and peaks might be attributed to the branch of starch and methyl cellulose [5,30]. The average roughness (*S*_q_) increased with the increase of ZIF-8 content, which was evidenced in SEM images before. The limited compatibility between blend and ZIF-8 nanoparticles might be responsible for this phenomenon. From the value of *S*_sk_ we could conclude that there were more hollows than peaks since they are negative value, which prefers the film possess a relatively smooth surface. With the content of ZIF-8 increased, the *S*_sk_ value increased and revealed the increase of roughness. Hence, the results of SEM and AFM consistently indicated that the roughness of films increased with the content of ZIF-8 increase [26,28,37].

### 3.3. Mechanical Properties

The values represent mean of five replicates. 

The methyl cellulose has generated great interests due to its excellent mechanical property as a source of fillers. The study investigated the effect of different mass fractions of methyl cellulose (0, 1, 3, 5 and 7 wt. %) on the tensile strength and elongation at break of the PVA/starch blend membranes. The result was shown in Table 2. It could be seen that the tensile strength decreased slightly when the mass fraction of methyl cellulose was 1%. It was because such a low concentration of methyl cellulose was deemed as an impurity which destroyed the homogeneity of the film [5]. Then the tensile strength of the film increased from 4.73 to 7.99 MPa as the content of methyl cellulose increased from 1% to 5%, due to the improvement of the interactive force between the methyl cellulose, starch and PVA macromolecule. However, the tensile strength decreased again when the addition of the methyl cellulose increased continuously. This was attributed to the slight aggregation of methyl cellulose at such concentration [7,38]. The effect of different concentrations of methyl cellulose on the elongation at break was similar to the tensile strength [39]. It can be seen from Table 1 that the maximum 505% was reached when the methyl cellulose was 5%. Thus, it can be concluded that composite with addition of 5 wt. % methyl cellulose gives the best mechanical property with maximum tensile strength and a satisfying elongation at break. The PVA-S-MC5% was then modified by different concentrations (0.01, 0.05, 0.09 wt. %) of ZIF-8 nanoparticle for further study.

As shown in Table 2, a slight decrease of tensile strength was observed as 0.01 wt % ZIF-8 nanoparticle was added to PVA-S-MC5%. The reason was similar to the explanation of 1 wt % methyl cellulose was introduced. The tensile strength of the nanocomposite film increased to 8.92 MPa as the concentration of the ZIF-8 was 0.05%. That was because there are more ZIF-8 nanoparticles distributed between the macromolecules, which enhanced the intermolecular interaction including hydrogen bonding and chemical bonding [40]. Moreover, the rigid structure of the ZIF-8 also elevated the chain strength of the polymer [41]. As the content of ZIF-8 increased continuously, the tensile strength of the membrane decreased again and even lower than the PVA-S-MC5%. This could be attributed to the agglomeration and high porosity of ZIF-8 nanoparticles, which largely degrade the homogeneity of the nanocomposite membrane [32]. Different from the tensile strength, the elongation at break of the composite membrane increased with the increasing of ZIF-8 content. This could be attributed to the inherent structure of the amorphous region of the polymer chain was destroyed as ZIF-8 nanoparticles were introduced to the membranes. The steric hindrance of ZIF-8 disrupted the ordered arrangement of the polymer chains and enhanced the fluidity of the chain [42].

### 3.4. Thermal Stability Analysis

**Figure 6:**
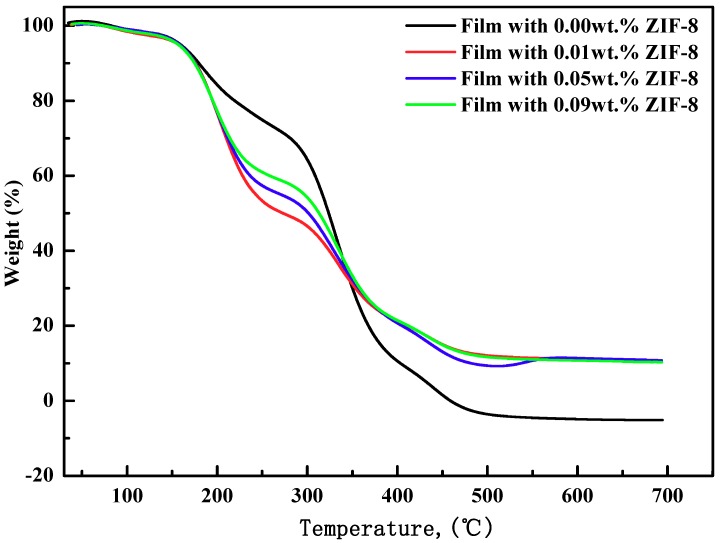
TGA characterization of PVA/starch/MC blend films with different concentration of ZIF-8 nanoparticle.

TGA analysis was carried out to explore the thermal stability of the nanocomposite membranes with different concentrations of ZIF-8. As shown in Figure 6, the thermal decomposition process of four samples mainly contains four stages. The slight weight loss between 100 to 150 °C corresponded to the unconjugated and chemically combined water in the films. When temperature raised from 150 to 300 °C, the weight losses of the nanocomposite membranes with different concentrations of ZIF-8 were equivalent. Nevertheless, the PVA-S-MC5% exhibited lower weight loss during this period. This is due to the decompositions of the branched-chains of the polymer, hydroxyl, and the residual solvent in the pores of ZIF-8 nanoparticles [35,42]. From 300 to 420 °C, there was a heavyweight loss because of the thermal decomposition of PVA, starch, and methyl cellulose and the volatilization of the polymer products [10,31,35,38,43]. The result was opposite in the last stage, the films with ZIF-8 nanoparticles shown a lower weight loss during this period. This could be attributed to the porosity of ZIF-8, and the interfacial gap between ZIF-8 and membrane decreased the heat transfer properties and hindered the evaporation of the decomposition products [27,32]. When the temperature is raised above 420 °C, ZIF-8 decomposed and generated ZnO. The film further decomposed into charcoal [27,32,33]. In general, the concentration of ZIF-8 had little effect on the thermal stability of film, but the addition of ZIF-8 could obviously improve the thermal stability and mechanical properties of the films.

### 3.5. Moisture Absorption

**Figure 7:**
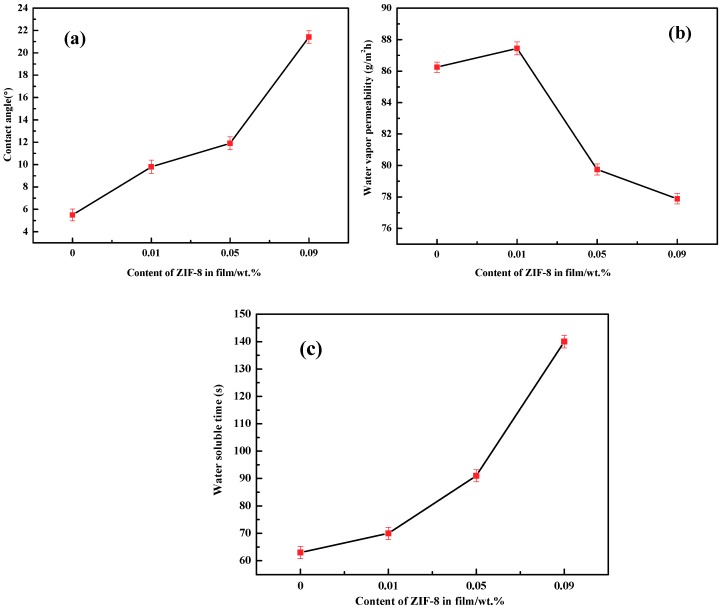
Contact angle (**a**), water vapor permeability (WVP) (**b**) and water solubility (WS) (**c**) of PVA/starch/MC blend films with different concentration of ZIF-8 nanoparticles.

Figure 7 demonstrated that the PVA-S-MC5% has an extremely hydrophilic surface with a contact angle of 4.52°. The hydroxyls existed in abundance in PVA, starch, methyl cellulose, and plasticizer accounted for this result [5,44,45]. The high polarity of the hydroxyl group also led to the relatively high water vapor permeability and short water-soluble time. After different contents of ZIF-8 were introduced into the film, the hydrophobicity of the membranes enhanced as indicated by the increase of contact angle [26,35,36,42,46] and water-soluble time as well as the decrease of water vapor permeability. The results were caused by the super-hydrophobic and relatively rigid structures of ZIF-8 nanoparticles. The hydrophobicity of ZIF-8 prevented the diffusion of water molecules in the films, and the rigid structure allowed films possess better resistant to dissolve in water. On the other hand, according to the SEM photograph, the roughness of membrane increased after the incorporation of ZIF-8. The larger roughness of the film also caused the increment of the contact angle [18]. Overall, the hydrophilicity of the nanocomposite membranes were improved after the addition of ZIF-8 nanoparticles, which is beneficial to its application in the high humidity environment.

### 3.6. Barrier Properties

**Figure 8:**
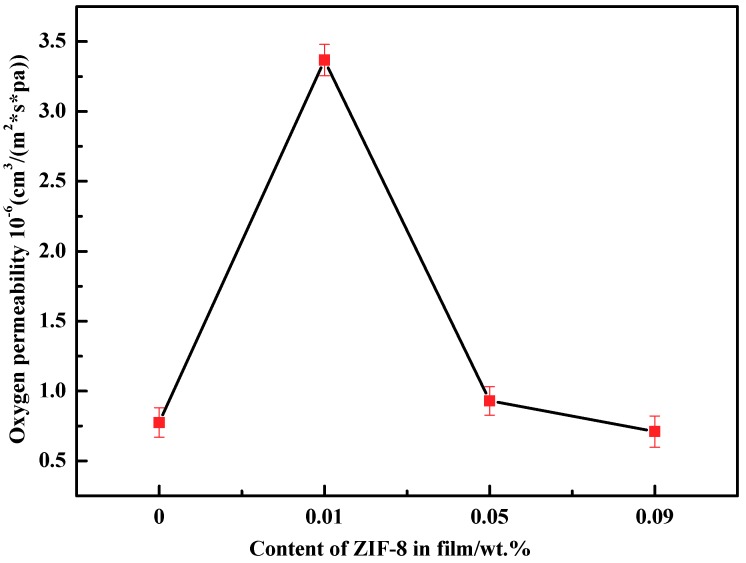
The oxygen permeability of PVA/starch/MC blend films with different concentration of ZIF-8 nanoparticle.

The oxygen permeability of films with different contents of ZIF-8 is shown in Figure 8. In the case of 0.01% ZIF-8, the oxygen permeability increased by 334.58% compared with the PVA-S-MC5%. PVA is a good barrier against oxygen and the PVA based membranes usually had low oxygen permeability as the result in our study. After ZIF-8 was introduced into the blend, the high porosity in nanoparticles and voids between ZIF-8 and polymer matrix provided channels for gas molecules to migrate through the film [32,46]. In addition, the destructive action of ZIF-8 on the amorphous region of the polymer enhances the mobility of the polymer chain and the roughness of the membrane, providing more effective area of the membrane and the possibility for gas molecules to pass through the chain gap [28,42]. When ZIF-8 content increased to 0.05% and 0.09%, a rapid decline of OP appeared and the value was close to PVA-S-MC5% which was 0.7 × 10^−6^ cm^3^/(m^2^ s pa). The possible reason should be that mounts of ZIF-8 deposited evenly in the gap between the polymers and blocked the channel of gas molecules.

### 3.7. UV–Visible Spectroscopy Analysis of Nanocomposite Films

**Figure 9:**
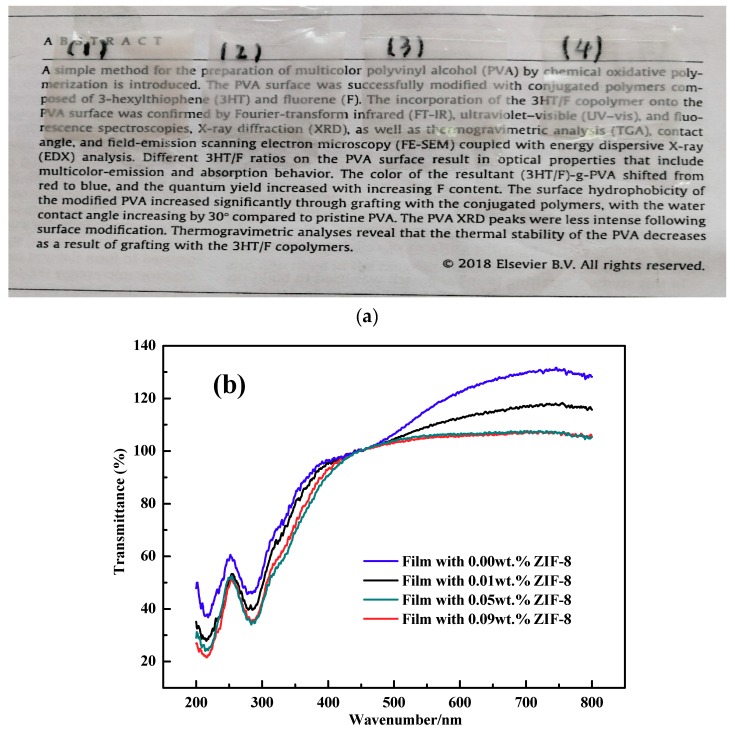
(**a**) The digital photos of films: (1) film with 0.00wt.% ZIF-8; (2) film with 0.01 wt. % ZIF-8; (3) film with 0.05wt.% ZIF-8; (4) film with 0.09 wt. % ZIF-8; (**b**) % transmittance of films.

The digital photos of films and UV-visible spectroscopy were shown in Figure 9. From Figure 9a it can be observed that all the films are transparent and the transparency was not affected after the addition of ZIF-8. The phenomenon can be attributed to the small size of ZIF-8 and their good dispersion in polymer [30]. Generally, the consumers prefer the packaging bag with high transparency which they could observe the food directly. On the other hand, the films with low UV-visible transmittance are good for foods since it can prevent lipids oxidation induced by visible light [39]. In our research, PVA-S-MC film possess highest transmittance, and the transmittance decreased with increase of ZIF-8 nanoparticles. The decrease of transmittance with addition of ZIF-8 was resulted from the low light transmission of particles and scattering of light [43]. The more ZIF-8 added, the lower the transmittance. Besides, relatively uneven distribution of ZIF-8 in polymer when its content increased to 0.05% and 0.09% also caused decrease of transmittance. The decrease of transmittance is beneficial for food package.

## 4. Conclusions

In this work, we have successfully synthesized ZIF-8 nanoparticles with the diameter about 100 nm and introduced it into the PVA-S-MC5% films. The effects of the concentration of ZIF-8 on the performance of films were investigated. The tensile strength and elongation at break of the nanocomposite film reached the maximum as the content of the ZIF-8 was 0.05%. Meanwhile, the thermal stability of blend films improved with the addition of ZIF-8. The SEM and AFM indicated that ZIF-8 changed the agglomeration of the raw materials and increased the roughness of the membranes. Because of the component and porous structure of ZIF-8, the water-resisting property of the nanocomposite membrane increased as the content of ZIF-8 increases, which was embodied in the water-soluble time and water vapor permeability. The oxygen permeability increased first and then declined as a result of ZIF-8 blocking the gap between macromolecules which provide channel for gas molecule. In summary, it can be concluded that 0.05% ZIF-8 loaded PVA/starch/methyl cellulose blend film possess the optimum performance and expand the application of film in postharvest of fruits and vegetables.

## Figures and Tables

**Table 1:** Average thickness of films.

Film Composite	Thickness (um)
PVA-S	98.3 ± 0.23
PVA-S-MC 1%	97.9 ± 0.12
PVA-S-MC 3%	97.8 ± 0.14
PVA-S-MC 5%	97.5 ± 0.25
PVA-S-MC 7%	98.1 ± 0.14
PVA-S-MC5%-ZIF-8 0.01%	97.1 ± 0.16
PVA-S-MC5%-ZIF-8 0.05%	97.4 ± 0.27
PVA-S-MC5%-ZIF-8 0.09%	97.9 ± 0.19

**Table 2:** Mechanical properties of PVA/S/MC films and PVA/S/MC-ZIF-8 films.

Film Composite	Tensile Strength (MPa)	Elongation at Break (%)
PVA-S	5.36 ± 0.21 ^c^	225 ± 2 ^d^
PVA-S-MC 1%	4.73 ± 0.26 ^d^	291 ± 2 ^d^
PVA-S-MC 3%	6.42 ± 0.15 ^b^	335 ± 2 ^d^
PVA-S-MC 5%	7.99 ± 0.55 ^a^	505 ± 3 ^c^
PVA-S-MC 7%	6.62 ± 0.27 ^b^	442 ± 1 ^d^
PVA-S-MC5%-ZIF-8 0.01%	6.72 ± 0.16 ^b^	554 ± 3 ^c^
PVA-S-MC5%-ZIF-8 0.05%	8.92 ± 0.23 ^a^	636 ± 2 ^b^
PVA-S-MC5%-ZIF-8 0.09%	7.64 ± 0.19 ^a^	747 ± 3 ^a^

Different letters within the same column indicate significant differences (*p* < 0.05).
